# Relationships between work ethic and motivation to work from the point of view of the self-determination theory

**DOI:** 10.1371/journal.pone.0253145

**Published:** 2021-07-01

**Authors:** Damian Grabowski, Agata Chudzicka-Czupała, Katarzyna Stapor

**Affiliations:** 1 Faculty of Psychology, Department of Social and Organizational Behavior, SWPS University of Social Sciences and Humanities, Katowice, Poland; 2 Faculty of Automatic Control, Electronics and Computer Science, Silesian University of Technology, Gliwice, Poland; University of Texas at Arlington, UNITED STATES

## Abstract

Most studies on motivation to work concentrate on its environmental and situational antecedents. Individual values are not the point of interest of empirical analyses. The aim of the research described in the paper was to seek possible relationships between work ethic and motivation to work. A hypothesis was put forward that work ethic, in the classical Weberian approach, is connected with motivation to work, from the point of view of Ryan’s and Deci’s self-determination theory. The study on a sample of 405 Polish employees was conducted with use of the Polish version of *Multidimensional Work Ethic Profile* MWEP-PL and *Work Extrinsic and Intrinsic Motivation Scale*, in the Polish adaptation WEIMS-PL. The Canonical Correlation Analysis was used to assess the simultaneous interrelationships between two sets of the variables measured. The results show that selected dimensions of work ethic, such as centrality of work, valuing hard work, perceiving work as an obligation, anti-leisure sentiment and delay of gratification are positively related to autonomous dimensions of motivation: intrinsic motivation, integration and identification, and non-autonomous introjection. Attributing a high value to hard work, including the conviction that it leads to success, aversion to wasting time and self-reliance correlate positively with taking up work for extrinsic rewards and with the desire to acquire a positive opinion about oneself as well as gain approval and recognition from others. Work ethic is connected on the one hand with autonomous motivation, including in particular intrinsic motivation, and on the other hand with extrinsic motivation, with the striving for success, which is the result of work. After empirical verification the findings could become a base for training programs and shape the way of influencing people’s motivation, morale, attitude towards work and job satisfaction. They can result in the way employees are managed and selected for different tasks.

## Introduction

Most empirical studies on work motivation and occupational behavior focus on the importance of environmental and situational characteristics such as working conditions and pay, organizational structure, job characteristics, task characteristics, working time flexibility, role of the manager and being subject to the latter’s control, as well as organizational climate [1–4]. Research also relates motivation to stressful environmental factors [1]. Some researchers point out that the external context in which an individual performs a task influences the intrinsic motivation to perform it, which may contribute to creative achievements [5]. Research was also conducted on motivational potential of meaningful work [6]. Some studies show how work-related and individual factors are related to psychological work ability and job mobility motivation in specific age, e.g. in later adulthood [7]. The relationships between motivation to work on the one hand and satisfaction with its performance and occupational burnout on the other hand was subject of studies as well [8].

Our review shows that research is still lacking that would connect individual predisposition or values subscribed to with motivation to work. The few empirical analyses carried out in this area prove the existence of relationships between affective organizational attachment, interest in work, acceptance of risk connected with its performance, perceived own competences and motivation to work [9], as well as between locus of control and motivation [10], and between agreeableness, conscientiousness, commitment to work, including attributing a high value to work, and motivation to learn, supposed to improve the quality of work [11].

Although the number of studies linking beliefs and values to motivation is not large, many scholars clearly pointed to the existence of interrelationships. Rokeach [12,13] has already presented values and beliefs as an inseparable element of motivation. Similarly, Lewin [14] considered values to be an important “guides” of behavior, because they trigger the goals to which one aspires. The self-concordance model of motivation [15] suggests that people are more inclined to pursue goals consistent with their autonomous values. The authors of this model, which measures intrinsic motivation, are guided by the assumption that people are intrinsically motivated by goals that result from the values they hold in high regard.

Studying motivation to work in the context of work-related values and work-related beliefs is rare [11]. There is a particular lack of research on the relationships between work ethic, understood as a multidimensional attitude towards work, which is a value in itself, and intrinsic and extrinsic motivation described by the self-determination theory. Few analyses point to the existence of certain relationships between the components of work ethic and intrinsic motivation, although work ethic in this case was studied in an Islamic version. However, study by Hayati and Caniago [16] on the Islamic work ethic and its relation to intrinsic motivation treated work ethic and intrinsic motivation as single dimension. Many studies highlight the importance of work ethic in business and in the capitalist economy [17–19]. This study aimed to check for potential relationships between the motivation to work and the dimensions of work ethic, reflecting human beliefs concerning work, the attitude towards being rewarded for work, leisure time, or the ability to rely on oneself in various activities, was designed to fill the gap in research into the area. We believe it is important for both cognitive and practical reasons to find an answer to the question as to whether any relationships between the variables mentioned above exist. Finding out about the strength and direction of these relationships may help to make a more effective impact on employees, to increase their motivation enabling them to act effectively and to achieve self-satisfaction and job satisfaction. It may also make it possible to prepare professionals better for training interventions. Knowledge about the relationships between the different components of both variables makes it possible to obtain better insight into the meaning of an individual’s autonomous values, attitudes, beliefs and needs, as well as into the nature and sources of their motivation. The findings presented in this study are exploratory in nature and their effects may require the construction of a more extensive model of dependencies. In fact, we do not know if and how work ethic, understood as a syndrome of different attitudes and beliefs about work, is connected with motivation to work.

### Work ethic as a system of attitudes and beliefs

Work ethic means attributing value to hard work and industriousness, stigmatizing idleness, fulfilling the obligations, and the belief that work should be done in the best possible way [20]. To fulfill the obligations means here a moral duty, while industriousness is considered a virtue, i.e. a desirable moral quality [21]. This term describes the cult of work, manifested in the respectful treatment of, or even reverence for work [22]. Work ethic involves perceiving and treating work as a duty or obligation and as a moral value. It consists of norms, prohibitions and orders, beliefs, attitudes and behaviors, both desirable and undesirable, connected with work valuation [20,23].

In the psychological sense, work ethic is a syndrome of attitudes and beliefs, with strongly outlined emotional-judgmental components. Miller [24] described seven dimensions of the syndrome, on the basis of analyses by Furnham [21]:

1. Belief in the sense of hard work, the conviction that it leads to success and that it is a recipe for problems and difficulties in life;
2. Centrality of work, the conviction that it is the basic activity in life–“but the most important thing was that even beyond that labor came to be considered in itself the end of life”;
3. Distaste for wasting time, tendency to treat time as a valuable resource–“waste of time is thus the first and in principle the deadliest of sins”;
4. Distaste for leisure, i.e. the conviction that free time activities are less valuable: “not leisure and enjoyment, but only activity serves to increase the glory of God”;
5. Delay of gratification, recognizing the value of rewards one has to wait for “the idea of expectant waiting for the Spirit to descend”), with importance attached also to work without rewards–the assumption that work in itself is a reward;
6. Independence, self-reliance at work, individualism;
7. Morality and ethics, i.e. placing emphasis on honesty in relationships with others, the assumption that honest conduct should be the content of the work [20] (p. 96–105).

These components can be put in order and structured. The core of a high work ethic is the conviction that work is a central value in life, so it should be done in a perfect and honest manner. Doing work well means devoting a considerable amount of effort and sufficient time to it. Therefore, the components of work ethic are deemed to include the requirement to save time, reduce leisure time, as well as the precept not to consume rewards, as they change people’s attitude towards other values. Also, worth mentioning are new research results on studies regarding the relationship of ethical culture and leadership with employees’ innovation [25,26].

### Work ethic and motivation to work. Self-determination theory

In the concept of work ethic, one can see descriptions of energy-related components, such as the requirement to increase effort and the high value given to it, i.e. emphasis on the importance of hard work. A job well done is also an efficient and effective action. The conceptualization and operationalization of work ethic performed by Mann [27] emphasize the importance of striving to improve oneself, looking after the quality of work and persistently pursuing of goals, i.e. factors which may be associated with motivation. Work ethic, by underscoring the importance and strengthening the training of independence, also triggers the motivation to achieve, conducive to economic development [21], and therefore these variables can be interrelated. Few studies also indicate the relationship between the work ethic syndrome and intrinsic motivation [16].

Cassidy and Lynn [28], in their conceptualization of achievement motivation, treat work ethic, defined as the performance of work for the sake of work itself, the desire to work hard and to derive satisfaction from such activity, as a component of motivation. Ethic understood in this way is placed here alongside other components of motivation, such as the desire to have and earn money, the need for dominance/power, the pursuit of perfection, the desire to achieve high standards, the tendency to compete and to perform better than others, as well as the desire to achieve a high status and prestige. Among the dimensions of motivation, the authors mentioned above also list the tendency to achieve mastery, which they understand as focusing on new challenges and situations that require one to master new skills.

Story and colleagues [29] suggested that work ethic, striving for perfection and mastery should be treated as components of the intrinsic motivation to achieve, while striving to have and to earn, the need for dominance, striving to compete and the desire to gain prestige should be treated as the extrinsic motivation to achieve. It should be noted, however, that in some samples an intrinsically motivating work ethic correlates with extrinsically motivating material needs, identified with earning money, the need for dominance and the need for prestige [28].

The division into intrinsic and extrinsic motivation has existed for a long time in the field of labor psychology, but only the emergence of a macro theory in the form of the self-determination theory [30] brought a new quality to research into work motivation. The self-determination theory, apart from the central division into autonomous and controlled motivation, postulates a multidimensional conceptualization of motivation. Ryan and Deci [30], assuming that each individual develops in relation to the actions he or she takes, and following many years of research, propose a macro theory which places emphasis mainly on the organic mechanisms of involving the internal resources of a human individual in his or her development, and more precisely in the development of personality and in self-regulation of behavior. According to these authors, the key process supporting the optimal functioning of people is their natural striving to improve and develop, manifested in the satisfaction of universal basic needs like social relationships and intimacy, competence and autonomy. They underline the role of behaviour in accordance with one’s own interests and values.

Autonomy understood in this way should not be confused with independence, although they may be interrelated. As a consequence, an individual satisfying such needs may feel pleasure and contentment. Research has shown that these needs are natural, but also that their properties are subject to situational influences that trigger intrinsic or extrinsic motivation, depending on the integrated orientation of the respective individual’s life goals [31–33].

The traditional conceptualization of intrinsic motivation assumes that this motivation refers to a situation in which behavior is triggered by different activities of the individual, interesting in themselves, causing spontaneous satisfaction and joy. At the same time, extrinsic motivation is clearly separated, as motivation triggering activities which are not interesting or satisfying in themselves for the individual, but which as a consequence lead to the valued effects. In this approach, extrinsic motivation is instrumental. In the context of work, however, extrinsic motivation has a dominant position and a wider range of types, contributing to the satisfaction of different needs, but according to Ryan and Deci [34], it is intrinsic, immanent motivation that represents the natural tendency an individual has to seek new challenges, learn and improve, based on enthusiasm, interests and passions. Intrinsic motivation understood in this way is a manifestation of a completely autonomous, self-determined, immanent motivation connected with the individual experiencing positive emotions [30,35,36]. The opposite of intrinsic motivation is extrinsic motivation in the form of external regulation, although in the self-determination theory there is also amotivation.

Amotivation is a state characteristic of non-autonomous behaviors, consisting in lack of regulation and reluctance to act. In the subject literature, it is compared to Seligman’s learned helplessness [37]. In the case of amotivation and external regulation, human behavior is completely independent of the individual, and it is controlled by external factors. Proper extrinsic motivation is a continuum of states regulated both extrinsically and intrinsically. It may vary in its intensity–from external regulation, through introjection and identification, to integration. Introjection is accompanied by involvement of the Ego, and behavior is partially controlled by the individual here, while in the case of identification and integration and of proper intrinsic motivation, the individual manifests fully autonomously regulated behavior. The differences in these three levels of motivation consist in the varying degree of internalization of values and goals underlying the behavior. Introjection is regulation consisting in taking action to gain self-approval and approval of those around the individual, for example by doing work to enhance one’s self-esteem, increase one’s prestige, and avoid shame. In the case of identification, the individual identifies with a set of values and meanings, accepting them as his or her own, while in the case of integration, the specific value or meaning becomes part of the system of definitions of the Self, creating the basis for autonomous regulation of behavior [38,39]. Therefore, identification and integration are still part of the system of extrinsic motivation, but one which is already regulated autonomously, and fully autonomous in the case of identification. The difference between autonomous regulation and integration, in the case of intrinsic motivation, boils down to activation of emotions, and in the case of integration–to cognitive activity [40].

Autonomous regulation, referring to intrinsic motivation, integration and identification, is associated with qualities such as resourcefulness and courage. Controlled regulation, i.e. introjection and external regulation mechanisms, provides the basis for industriousness, regularity, perseverance, strong will and prudence. Striving to improve oneself and implementing standards leading to an ideal image of the self represents the autonomous regulation perspective, while striving to achieve what should be achieved according to others is a manifestation of controlled regulation [33].

Work ethic involves both resourcefulness and industriousness, as well as prudence [21] and the realization of a perfect image of oneself [27]. Hence, it may be assumed that work ethic as a syndrome of beliefs which value work is associated both with autonomous motivation and with controlled, non-autonomous regulation. Traditionally, in line with the definition of work ethic, work means coercion and obligation. However, the definition of work ethic also implies the importance of individual independence, the need to rely on oneself and to strive to achieve [21]. Recent conceptualizations of work ethic also include the pursuit of excellence and mastery, which guarantee high-quality work [27].

The findings of the studies by Cassidy and Lynn [28] quoted above showed that intrinsically motivating work ethic correlates with extrinsically motivating needs, such as earning money and striving for dominance and prestige. These findings are also consistent with the research conducted by Wollack [41], in which it turned out that work ethic referred to the attitude towards pay, i.e. attributing a high value to earning money at work. The research also proved the existence of links between work ethic and social status, defining one’s position among the others and both self-perception of this status and the perception of that status by the social environment, friends, relatives and co-workers, which is associated with prestige. The work ethic conceptualization built by Wollack [41] also includes the pursuit of promotion. Status, prestige and pursuit of promotion are connected with introjection, and earning money is connected with external regulation.

Finally, some recent developments in SDT theory should be cited. In [42] the Authors studied public employee’s motivation for a public service career and developed a SDT-based measurement instrument that captures different motivations for it. A meta-analytic review [43] of almost 100 studies examining the antecedents and consequences of basic need satisfaction at work provides interesting and new contributions and challenges to the SDT literature. Through the lens of SDT in [44] the Authors tested the mediating effect of autonomy, how internal sources of innovations (i.e. emanating from an agency’s senior leadership/employee workgroups) affect employees’ job satisfaction.

Thus, if both work ethics and intrinsic motivation are associated with job satisfaction and innovation [21,25,44], it can be assumed that the work ethic and motivation also show significant relationships. The important question is which components of ethics are most strongly associated with intrinsic motivation and which are weaker.

#### Research questions and hypotheses

We asked the research questions about the possible relationships between work ethic dimensions and the motivation to work, i.e. between autonomous and controlled regulation, and about the nature of them. Research questions were also put forward concerning the existence and strength of the relationship between work ethic dimensions and the individual methods of regulation, i.e. autonomous and non-autonomous regulation, as well as about whether and how work ethic dimensions correlate with amotivation.

On the basis of the considerations presented above, we hypothesize that:

H1. Positive relationships exist between the dimensions of work ethic (work as moral value and obligation, hard work, centrality of work, wasted time, anti-leisure sentiment, delay of gratification, self-reliance and morality/ethics) and autonomously regulated motivation (intrinsic motivation, integration, identification) as well as non-autonomous introjection.

H2. Positive relationships exist between the dimensions of work ethic that involve attributing value to success and to the ways of achieving it (work as moral value and obligation, wasted time and self-reliance) and non-autonomously regulated motivation (introjection and external regulation).

## Materials and methods

### Study sample and procedure

A quota sampling [1] being a non-probabilistic version of stratified sampling was used to obtain a sample of participants for our study. A population was first segmented into 4 sub-groups according to the size of employment (micro-enterprises, small, medium and large businesses) based on the structure obtained from the Central Statistical Office in Poland. Samples of participants were then selected from each subgroup based on the specified proportion [45].

The sample consisted of 405 individuals working in various organizations in southern Poland. The sample included 227 women (56%) and 178 men (44%). The study covered a group of people aged 19 to 71. The average age of the respondents was over 35.23 (*SD* = 12.05, *Range* = 19–71) years. The sample included people with different educational backgrounds. The largest number of respondents had secondary education (194 individuals, 48% of the sample), higher education (160 individuals, 39% of the sample) and vocational education (51 individuals, 13% of the sample). The study covered 90 individuals working in micro-enterprises (employing up to 9 people) (22% of the sample), 107 employees of small businesses, employing up to 49 people (26% of the sample), 84 employees of medium-sized businesses employing up to 249 people (21% of the sample), and 124 employees of large businesses (employing over 250 people) (31% of the sample).

The study subjects included individuals pursuing different professions (administrative support (105 individuals, 26% of the sample), accounting/financial (95 individuals, 23% of the sample), technology (105 individuals, 26% of the sample), health/safety (100 individuals, 25% of the sample)) and employed in various industries (manufacturing (150 individuals, 37% of the sample), services (130 individuals, 32% of the sample), retail (125 individuals, 31% of the sample)). The majority of the study subjects (283 individuals, 70% of the sample) worked under an employment contract, full-time, 57 individuals (14% of the sample) were self-employed, and 65 individuals (16% of the sample) worked under civil law contracts. The majority were employees of businesses with nationwide reach (302 individuals, 75% of the sample), while the remaining group of 103 individuals (25% of the sample) worked in companies with international reach. The average length of service being 12.94 (*SD* = 11.64, *Range* = 0.5–45) years.

Efforts were made to examine people of different ages, both women and men, employees working for a given company for at least six months in various industries.

The research was conducted in 2018, from June to December. The respondents did not receive any remuneration for their participation in the survey and filled out a set of questionnaires using the paper and pencil form.

The research was conducted in compliance with the ethical standards in line with the provisions of the Declaration of Helsinki. The Departmental Research Ethics Committee of Faculty of Psychology at SWPS University of Social Sciences and Humanities (Katowice, Poland) (Ref. number: WKEB63/05/2020/Human participants, project title: Relationships between work ethic and motivation to work from the point of view of the self-determination theory) approved the research proposal and the consent procedure. The respondents agreed to participate voluntarily, they were informed about its purpose, assured about its complete anonymity, and obtained information about the possibility of withdrawing from it at any time.

### Measures

To measure work ethic we used the Polish version of Multidimensional Work Ethic Profile (MWEP), an abridged version of the MWEP questionnaire created by Miller [24], adapted by Grabowski and Chudzicka-Czupała [23,46], and abridged by Grabowski [47]. The questionnaire is composed of 35 items and 7 scales (or 7 subscales) (with 5 items in each scale), which correspond with 7 dimensions of work ethic: belief in the sense of hard work (Hard work), Centrality of work, distaste for wasting time (Wasted time), distaste for leisure (Anti-leisure sentiment), Delay of gratification, Self-reliance and morality and ethics (Morality/Ethics). Five statements were added to the list of 35 items mentioned above, related to the conviction that work is a value and a moral obligation (Work as moral obligation—WMO scale).

Participants indicated their attitudes toward statements using a 1 (“I strongly disagree”) to 5 (“I strongly agree”) scale. Statistical analyses also used an index constituting the sum of all the seven subscales, i.e. MWEP-total, without the WMO scale.

To study motivation to work, the Work Extrinsic and Intrinsic Motivation Scale (WEIMS) was used, built by Canadian psychologists [48], in the Polish adaptation by Chrupała-Pniak and Grabowski [49]. Both the original tool and the Polish adaptation demonstrate satisfactory psychometric properties. The scale represents an operationalization of the individual regulations of motivation, taken into account in the self-determination theory, i.e. intrinsic motivation, integration, identification, introjection, external regulation and amotivation. The original tool consists of 18 items, with 3 scale items corresponding to each of the six regulations (six scales or subscales of WEIMS). A 24-item method was used in the study, with one statement added to each scale (subscale).

The respondents’ task was to take a position on the items using a seven-point scale from 1 to 7 (with 1 meaning “This statement doesn’t describe me at all”, 3 –“This statement describes me in rather moderately”, 7 –“This statement describes me absolutely accurately”).

The Work Self-Determination Index (WSDI) was also used in the calculations. This index is calculated using the following formula: -3*amotivation + -2* external regulation + -1*introjection + 1*identification + 2*integration + 3*intrinsic motivation; and it simply means the degree of self-determination of behavior at work [48,49].

Table 1 presents descriptive statistics and the reliability coefficients i.e. Cronbach’s alpha and McDonald’s omega of Multidimensional Work Ethic Profile (MWEP), Work Extrinsic and Intrinsic Motivation Scale (WEIMS) subscales and global indices (MWEP, the sum of 7 dimensions, Work Self-Determination Index WSDI).

**Table 1:** Descriptive statistics and reliability coefficients of work ethic dimensions (MWEP) and components of motivation to work (WEIMS).

	*M*	*SD*	*Minimum*	*Maximum*	*α*	*ω*
**Independent variables:**						
**1. Work as moral obligation**	17.86	3.81	5	25	0.73	0.74
**2. Hard work**	17.27	4.15	5	25	0.77	0.78
**3. Centrality of work**	18.11	3.93	5	25	0.70	0.71
**4. Wasted time**	18.17	3.56	5	25	0.64	0.65
**5. Anti-leisure**	13.76	3.93	5	25	0.73	0.75
**6. Delay of gratification**	17.12	3.94	5	25	0.70	0.73
**7.Self-reliance**	19.27	3.82	5	25	0.79	0.80
**8. Morality/Ethics**	20.90	3.57	5	25	0.69	0.71
**9. MWEP**	124.60	15.38	35	175	0.66	0.67
**Dependent variables:**						
**10. Amotivation**	11.26	4.25	4	28	0.55	0.61
**11. External regulation**	19.68	5.65	4	28	0.87	0.87
**12. Introjection**	18.24	5.03	4	28	0.73	0.75
**13. Identification**	15.45	6.17	4	28	0.86	0.86
**14. Integration**	16.98	6.46	4	28	0.89	0.89
**15. Intrinsic motivation**	18.96	5.64	4	28	0.85	0.86
**16. Work self-determination index**	14.93	32.62	-65	114	0.70	0.70

*M* = Mean value, *SD* = Standard deviation, MWEP–Multidimensional work ethic profile, *α* = Cronbach’s *α*, *ω* = McDonald’s *ω*—reliability coefficients.

The amotivation scale obtained a lower Cronbach’s α value in these studies, just like in previous studies, by the way, both on Polish and on Canadian samples [48], and its revision should be considered in the future.

The validity of the modified WEIMS version, which includes 24 items, was also checked by means of confirmatory factor analysis. A confirmatory factor analysis demonstrated that the WEIMS scale achieved satisfactory measures of fit of the six-factor model to the data (comprising four positions in each scale): *χ2* (df) = 789.49 (237), *RMSEA* = 0.076, *CFI* = 0.96, *sRMR* = 0.071, *NFI* = 0.95 [49,50].

#### Data analysis

Descriptive statistics, reliability coefficients and correlations were calculated with JASP (v0.12.2), a Confirmatory Factor Analysis (CFA) conducted using JASP (v0.12.2) and the Lisrel (v9.2) software, and Canonical Correlation Analysis (CCA) was conducted using STATISTICA (v12.0).

#### Canonical correlation analysis

We used the multivariate statistical method, Canonical Correlation Analysis (CCA) [51,52] to verify the two hypothesis and to investigate the magnitude and sign of the relationships between two sets of variables, one comprising the dimensions of work ethic construct and referred to as independent variables, and the second composed of factors from work extrinsic and intrinsic motivation, considered here as dependent variables.

The main goal of CCA is an assessment of the simultaneous interrelationships between two sets of variables. CCA focuses on the correlation between two new synthetic variables, called *canonical variates*, one is a linear combination of variables from the first set and the other is a linear combination of the variables of the second set. CCA constructs a *canonical function* that maximizes the *canonical correlation coefficient* which measures the strength of the overall relationship (correlational) between the two canonical variates. CCA develops multiple canonical functions, each is independent from the other canonical functions so that they represent different relationships found among the sets of dependent and independent variables. Each canonical variate is interpreted with *canonical loadings*, the correlation of the individual variables and their respective variates. *Redundancy index* is an amount of variance in a canonical variate (dependent or independent) explained by the other/opposite canonical variate in the canonical function. These may be summed to reveal an *overall redundancy index*.

## Results

### Preliminary analyses

Table 2 presents the correlation coefficients between individual dimensions of work ethic and motivation together with global indices (MWEP, the sum of 7 dimensions, Work Self-Determination Index WSDI).

**Table 2:** Correlations between dimensions of work ethic and components of motivation to work.

	1	2	3	4	5	6	7	8	9	10	11	12	13	**14**	**15**
**1. Work as moral obligation**	—														
**2. Hard work**	0.40***	—													
**3. Centrality of work**	0.40***	0.44***	—												
**4. Wasted time**	0.34***	0.43***	0.41***	—											
**5. Anti-leisure**	0.16**	0.24***	0.34***	0.15**	—										
**6. Delay of gratification**	0.22***	0.40***	0.25***	0.31***	0.08	—									
**7.Self-reliance**	0.10	0.11*	0.27***	0.25***	-0.09	-0.06	—								
**8. Morality/Ethics**	0.15**	0.02	0.28***	0.19***	-0.04	0.07	0.45***	—							
**9. MWEP**	0.44***	0.68***	0.75***	0.68***	0.43***	0.52***	0.47***	0.47***	—						
**10. Amotivation**	0.05	0.06	-0.06	-0.02	-0.01	-0.05	-0.03	-0.16**	-0.06	—					
**11. External regulation**	0.11*	0.19***	-0.01	0.16**	-0.04	0.08	0.17***	0.04	0.15**	-0.10	—				
**12. Introjection**	0.30***	0.36***	0.43***	0.25***	0.20***	0.22***	0.12*	0.16**	0.44***	0.03	0.21***	—			
**13. Identification**	0.20***	0.28***	0.37***	0.24***	0.12*	0.14**	0.14**	0.14**	0.36***	0.04	0.25***	0.67***	—		
**14. Integration**	0.20***	0.31***	0.48***	0.17***	0.23***	0.16***	0.04	0.15**	0.39***	0.04	0.04	0.65***	0.76***	—	
**15. Intrinsic motivation**	0.23***	0.34***	0.50***	0.23***	0.23***	0.24***	0.09	0.25***	0.47***	-0.11*	0.09	0.70***	0.61***	0.70***	—
**16. Work self-determination index**	0.13**	0.20***	0.48***	0.15**	0.22***	0.18***	0.02	0.24***	0.37***	-0.40***	-0.23***	0.51***	0.60***	0.77***	0.82***

MWEP–Multidimensional work ethic profile.

***p < .001

**p < .01

*p < .05.

It follows from the Table 2, as expected, that dimensions of work ethic are positively correlated, weak (about 0.1), moderate (0.2 or 0.3) and average (0.4) with motivation that is regulated autonomously (identification, integration and intrinsic motivation) as well as the non-autonomous introjection. The strongest correlations of the mentioned regulations exist with the Centrality of work, the moderate—with the Work as moral obligation, Hard work and Anti-leisure. Amotivation is correlated with the dimensions of work ethic very weakly, rather negatively and not significant, except from Morality/ethics.

### Results of Canonical Correlation Analysis (CCA)

Table 3 presents the results of the CCA. The results of tests of significance prove that only the first two canonical functions ((U1, V1), (U2, V2)) were statistically significant with *p*-values < 0.001 of the testing procedure of the canonical correlations (as implemented in STATISTICA package). The independent canonical variates U1, U2 are linear combinations of variables from the first set of variables defining work ethic construct, while the canonical variates V1, V2 are linear combinations of variables from the second set of variables defining work motivation (see Table 3).

**Table 3:** Work ethic and motivation. Results of CCA for two canonical functions: canonical correlations, loadings, shared variance and redundancy analysis of independent and dependent canonical variates.

**Independent variables**	**Canonical loadings of independent variates**	
	U1	U2
**1. Work as moral obligation**	0.412	0.482
**2. Hard work**	0.585	0.619
**3. Centrality of work**	0.920	0.028
**4. Wasted time**	0.362	0.531
**5. Anti-leisure**	0.448	-0.102
**6. Delay of gratification**	0.405	0.239
**7.Self-reliance**	0.098	0.532
**8. Morality/Ethics**	0.395	0.005
**Shared variance**	0.253	0.156
**Redundancy index**	0.087	0.021
**Canonical correlations**	0.585***	0.362***
**Overall redundancy (dependent set)**	0.182	
**Dependent variables**	**Canonical loadings of dependent variates**	
	V1	V2
**9. Amotivation**	-0.136	0.287
**10. External regulation**	0.000	0.807
**11. Introjection**	0.782	0.451
**12. Identification**	0.620	0.382
**13. Integration**	0.858	-0.003
**14. Intrinsic motivation**	0.931	0.122
**Shared variance**	0.436	0.183
**Redundancy index**	0.149	0.024

***p < .001.

To assess the contributions of the variables defining work ethic and motivation canonical variates in the canonical correlation, we used canonical loadings (assuming here that a loading greater than 0.4 proves that correlation between a corresponding variable and a variate is significant).

The first canonical correlation between the independent variate U1, being a linear combination of work ethic variables and V1, the dependent variate—a linear combination of work motivation variables is quite strong and equals to 0.585.

In the dependent variate V1, the highest and positive canonical loadings had intrinsic motivation (0.931), integration (0.858), introjection (0.782) and identification (0.620). According to the self-determination theory, the intrinsic motivation, integration and identification constitute the components of an autonomous motivation. This fact supports naming the canonical variate V1 as the “*high autonomous motivation*”. Moreover, it should be seen a strong positive correlation of variate V1 with introjection (0.782), which means that autonomous motivation is associated with striving after self and other approbations, thereby being a sense of duty. It caused to name the variate V1 of the first canonical function, the “*high autonomous motivation and duty*”. It should be noted that an amotivation variable had low negative correlation with the variate V1 and an external regulation is not correlated with it. The corresponding to V1, dependent variate U1 we call “*centrality of work in life*” because its highest canonical loading is that of Centrality of work (0.920). The remaining variables from the first set of work ethic construct, except for Hard work (0.585), show almost half the correlations, although still high: Anti-leisure (0.448), Work as moral obligation (0.412), Delay of gratification (0.405).

The canonical variate U1 in the first canonical function, explains 25.3% of the variance in the set of work ethic variables, and the associated variate V1–43.6% of the variance in the set of work motivation variables (see Table 2 for the shared variances).The independent canonical variate U1 for work ethic in the first canonical correlation “*centrality of work in life*”->“*high autonomous motivation and duty*” explains almost 15% (14.9%) of the variance in the dependent set of variables from work motivation (see Table 3 for redundancy index in dependent set).

In summary, the results of this study allow for the conclusion that people with high autonomous motivation and conviction that work is a duty, treat the work as a central value in their life more often, while the remaining activities could be less important.

In the second canonical function, the correlation between the independent variate U2, being a linear combination of work ethic variables and the dependent variate V2—a linear combination of work motivation variables is somewhat weaker and equals to 0.362.

We call the canonical variate U2 “*hard work*” as the canonical loading of the variable hard work is the highest (0.619). Simultaneously, we observe quite strong canonical loadings from the following variables defining work ethic: self-reliance and wasted time (0.532 and 0.531, respectively). This is equivalent to a conviction that hard, intensive work, self-reliance and saving time lead to a success, or ensure a prosperity in life. The highest canonical loading in the second, dependent variate V2 had external regulation (0.807), which is equivalent to a regulation controlled by awards and penalties. Simultaneously, the lowest canonical loadings in the variate V2 come from intrinsic motivation (0.122) which is an evidence of an autonomous regulation. This fact allows to name the canonical variate V2 as “*external control*”. At the same time, there is quite strong correlation with the variable introjection (0.451), which means that hard work motivated by a wish to gain approval from others is connected with obtaining through a work such awards like money.

The canonical variate U2 in the second canonical function, explains 15.6% of the variance in the set of work ethic variables, and the associated variate U1–18.3% of the variance in the set of work motivation variables (see Table 2 for the shared variances). The independent canonical variate U2 for work ethic in the second canonical correlation “*hard work*” -> “*external control*” explains only 2.4% of the variance in the dependent set of variables from work motivation (see Table 3 for redundancy index in dependent set).

The overall redundancy of dependent set of variables is equal to 18.2%. This means that 18.2% of variance in work motivation variables can be explained by the whole set of independent work ethic variables (i.e. predictors).

## Discussion and conclusions

The main aim of this exploratory research was to determine whether any relationships could be found between work ethic dimensions and motivation to work described by the self-determination theory, i.e. relationships with autonomous and controlled regulation. These regulations characterize human activity during the performance of work.

The research findings show that there are positive relationships between work ethic on the one hand and autonomous motivation and striving for recognition (including recognition from other people and self-satisfaction) on the other hand. There is a significant positive correlation between the dimension of centrality of work on the one hand and autonomous motivation and duty on the other hand. In other words, individuals who insist on the centrality of work, who value it highly, also in the moral sense, and who are convinced of the value of hard work, are at the same time highly motivated to do work they find exciting, as a component of their identity. At the same time, these individuals are also convinced that work should be done well and accurately. On the one hand, they find work exciting, interesting and challenging, on the other hand they believe that one should strive towards mastery when performing it, and treat this as a duty.

Individuals displaying autonomous motivation at work may treat good performance of the latter as a duty. This is one of the possible interpretations of the relationship between autonomous motivation and non-autonomous introjection. It can also be noted that high scores on the introjection scale do not have to indicate only actions resulting from the desire to gain recognition. It may also be a result of the fact that individuals motivated to perform work autonomously satisfy their general need to have positive relationships with other people [29]. Striving to be recognized and respected by others is a way of satisfying this need, and at the same time achieving this proves that the duty has been performed well. In other words, high scores on the introjection scale can mean that the individual motivated to a large extent intrinsically wants to win interest and approval from the environment because of the good performance of tasks.

Research has also shown that high value given to hard work, the conviction that it leads to success, combined with the belief that one needs to rely on oneself and avoid being dependent on others, is at the same time associated with the will to work for material rewards and with the pursuit of approval. These are extrinsic factors that are important for the performance of work. Although surprising, this result is consistent with the classical Protestant work ethic approach, in which we find both encouragement to do work out of duty, because work is an obligation, and affirmation of the pursuit of success, positive valuation of extrinsic indicators of success, such as the desire to earn money [21]. This result is also consistent with the research by Cassidy and Lynn [26] and the earlier studies by Wollack [41]. On the basis of the results obtained, hypotheses 1 and 2 can be accepted. The results also show that amotivation correlates negatively and weakly with the dimensions of work ethic.

To recapitulate, individuals with high autonomous motivation, a high need for recognition, and high intensity of introjection treat work much more often as a central value in their lives, while other activities are less important for them. Performance of interesting work which they like most probably makes it easy for them to value it highly, which co-occurs with their intrinsic need to take up and do work and their desire to maintain a good opinion of themselves as an employee and at the same time gain a positive opinion of their environment. Autonomous motivation co-occurs with introjection. An individual with autonomous motivation, having a high intrinsic motivation, treats good performance of work as his or her duty. Secondly, interesting work can be a source of high status and prestige, which is associated with activity being driven by the motivation to gain approval. This is also in line with earlier research results [22,28].

High scores on the introjection scale may generally indicate the fact that individuals autonomously motivated to work satisfy the need for positive relationships with others and for gaining recognition from others. According to the self-determination theory, controlled regulation, including introjection and external regulation, means striving to satisfy the need for positive relationships with others and for competence. Autonomous regulation, apart from satisfying these two needs, also makes it possible to satisfy the need for autonomy [31]. Intrinsic motivation combines all these motives, including those assigned to the other types of motivation, i.e. striving for integration, identification, introjection and external regulation. Only amotivation, or impersonal regulation, points to a lack of desire to satisfy these three needs. Amotivation also demonstrates a relationship with extrinsic control, i.e. controlled regulation. The results of the study described here show that activity based on extrinsic rewards may lead to amotivation. In the results of canonical analysis, this is proven by the weak positive correlation between amotivation and external regulation, and more precisely with the extrinsic control factor [35,36,53].

The study has a few limitations. First is the Polish context of our research. Work is less valued in Poland than, for example, in the United States [46], but more than in other countries [21,22]. It can therefore be assumed that Poles may have a lower work ethic than the inhabitants of post-Protestant countries. That is why our findings may not be generalized to other cultural settings, particularly outside of the Eastern Europe.Only the cross-cultural study would make it possible to compare Polish employees’ responses with the attitudes of representatives from other countries. Another limitation is that the study was based on self-assessment questionnaires. Their use resulted from the lack of other tools for measuring the studied variables as well as from the nature and definition of these variables, based on subjective judgment. However, these were accurate and reliable tools. Only the operationalization of specific regulation styles may be considered questionable due to the high correlations of introjection with identification, integration and intrinsic motivation [43]. It should be recalled, however, that within the self-determination theory itself, intrinsic (autonomous) motivation is based on mechanisms reserved for controlled regulation. Autonomous motivation leads to the satisfaction of three basic needs, while controlled motivation leads to the satisfaction of two needs [31].

Although the respondents were assured of anonymity, the responses might also be falsified due to the effect of the study subjects responding in accordance with what they imagine to be the socially desirable content, which in turn may have affected the final results of the study. However, an attempt was made to counteract this phenomenon by providing appropriate instructions and by assuring the respondents about the complete confidentiality of the data.

The research methodology could be improved and broadened by adding qualitative methods such as interviews and analyses based on interpretative phenomenological analysis (IPA). This would provide a deeper insight into the respondents’ feelings and into their experiences, and could definitely expand knowledge about the relationships between work ethic and motivation. Further research should also focus on the importance of other variables. It is worth checking the level of selected variables that may be relevant here, such as e.g. temperamental and personality determinants, other psychological characteristics, or characteristics related to morality. It may also be significant to take into account simultaneously the characteristics of the working environment and the organizational climate. Another significant development might involve controlling for the level of the respondents’ satisfaction with their professional work. It would be worth comparing in the future the dependencies existing within the group of managers, entrepreneurs and non-managers, as one may expect that the relationships between work ethic, attitude towards work and motivation might be more distinct in individuals with considerable autonomy, and that senior-level managers or entrepreneurs with considerable freedom of action are less likely to be forced to act under coercion.

In future research, it would be worthwhile controlling also for employee behavior that may be related to work ethic and result from motivation, or be connected to amotivation, such as civic organizational behavior, counterproductive behavior, and unethical pro-organizational behavior. Additionally, it could be interesting to consider the importance of work ethic and motivation to perform work in ethical or strategic decision-making within the company, e.g. in the way of implementing the CSR strategy, with simultaneous control for dispositional and environmental variables.

Research implications suggest that the findings may be important for the practice. We imagine workshops on work ethic and motivation, participation in which would let the individuals obtain better insight into the meaning of their own values, needs, and attitudes connected with work and into sources of their own motivation. It would be advisable to train individuals by focusing on the strengthening of their motivation, basing on their specific beliefs about work.

On the basis of the research findings, it can be assumed that a high work ethic characterizes more often individuals who display high intrinsic motivation, are motivated to perform interesting work, and strive to achieve high standards in it. The results may also point to the satisfaction of the need to have a positive opinion about oneself, as well as to the need for recognition and prestige, by individuals autonomously motivated to work. Attributing high value to hard work, the belief that it leads to success, and self-reliance are also related to the willingness to work for external, material rewards, and may result from the pursuit of positive relationships with others.

Research into the relationships between work ethic and motivation to work is in the exploratory phase, so both theoretical models and potential causal models require further empirical research. We firmly believe that despite these limitations and the lack of final theoretical conclusions, the research presented here contributes to a more complete understanding of human attitude towards work and points to important sources of motivation to work, as well as constitutes an important step towards building a more complete model of the interrelationships between the two variables.

## Supporting information

S1 Table Descriptive statistics and reliability coefficients of work ethic dimensions (MWEP) and components of motivation to work (WEIMS). *M* = Mean value, *SD* = Standard deviation, MWEP–Multidimensional work ethic profile, *α* = Cronbach’s *α*, *ω* = McDonald’s *ω*—reliability coefficients.
(DOCX)

S2 Table Correlations between dimensions of work ethic and components of motivation to work. MWEP–Multidimensional work ethic profile, ***p < .001; **p < .01; *p < .05.
(DOCX)

S3 Table Work ethic and motivation. Results of CCA for two canonical functions: canonical correlations, loadings, shared variance and redundancy analysis of independent and dependent canonical variates. ***p < .001.
(DOCX)

S1 File (XLSX)
