# Supplementary material for: Relationships between work ethic and motivation to work from the point of view of the self-determination theory
Source: PLoS One. 2021 Jul 1;16(7):e0253145. doi: 10.1371/journal.pone.0253145 (PMC8248600; doi:10.1371/journal.pone.0253145)
Supplement: S1 Table — M = Mean value, SD = Standard deviation, MWEP–Multidimensional work ethic profile, α = Cronbach’s α, ω = McDonald’s ω—reliability coefficients. (DOCX) [file pone.0253145.s001.docx]

**Table 1. Descriptive statistics and reliability coefficients of work ethic dimensions (MWEP) and components of motivation to work (WEIMS).**

|  | ***M*** | ***SD*** | ***Minimum*** | ***Maximum*** | ***α*** | ***ω*** |
| --- | --- | --- | --- | --- | --- | --- |
| **Independent variables:** |  |  |  |  |  |  |
| **1. Work as moral obligation** | 17.86 | 3.81 | 5 | 25 | 0.73 | 0.74 |
| **2. Hard work** | 17.27 | 4.15 | 5 | 25 | 0.77 | 0.78 |
| **3. Centrality of work** | 18.11 | 3.93 | 5 | 25 | 0.70 | 0.71 |
| **4. Wasted time** | 18.17 | 3.56 | 5 | 25 | 0.64 | 0.65 |
| **5. Anti-leisure** | 13.76 | 3.93 | 5 | 25 | 0.73 | 0.75 |
| **6. Delay of gratification** | 17.12 | 3.94 | 5 | 25 | 0.70 | 0.73 |
| **7.Self-reliance** | 19.27 | 3.82 | 5 | 25 | 0.79 | 0.80 |
| **8. Morality/Ethics** | 20.90 | 3.57 | 5 | 25 | 0.69 | 0.71 |
| **9. MWEP** | 124.60 | 15.38 | 35 | 175 | 0.66 | 0.67 |
| **Dependent variables:** |  |  |  |  |  |  |
| **10. Amotivation** | 11.26 | 4.25 | 4 | 28 | 0.55 | 0.61 |
| **11. External regulation** | 19.68 | 5.65 | 4 | 28 | 0.87 | 0.87 |
| **12. Introjection** | 18.24 | 5.03 | 4 | 28 | 0.73 | 0.75 |
| **13. Identification** | 15.45 | 6.17 | 4 | 28 | 0.86 | 0.86 |
| **14. Integration** | 16.98 | 6.46 | 4 | 28 | 0.89 | 0.89 |
| **15. Intrinsic motivation** | 18.96 | 5.64 | 4 | 28 | 0.85 | 0.86 |
| **16. Work self-determination index** | 14.93 | 32.62 | -65 | 114 | 0.70 | 0.70 |

*M* = Mean value, *SD* = Standard deviation, MWEP – Multidimensional work ethic profile, *α* = Cronbach’s *α*, *ω* = McDonald’s *ω* - reliability coefficients
