# Supplementary material for: Relationships between work ethic and motivation to work from the point of view of the self-determination theory
Source: PLoS One. 2021 Jul 1;16(7):e0253145. doi: 10.1371/journal.pone.0253145 (PMC8248600; doi:10.1371/journal.pone.0253145)
Supplement: S2 Table — MWEP–Multidimensional work ethic profile, ***p < .001; **p < .01; *p < .05. (DOCX) [file pone.0253145.s002.docx]

**Table 2.** **Correlations between dimensions of work ethic and components of motivation to work.**

|  | **1** | **2** | **3** | **4** | **5** | **6** | **7** | **8** | **9** | **10** | **11** | **12** | **13** | **14** | **15** |
| --- | --- | --- | --- | --- | --- | --- | --- | --- | --- | --- | --- | --- | --- | --- | --- |
| **1. Work as moral obligation** | — |  |  |  |  |  |  |  |  |  |  |  |  |  |  |
| **2. Hard work** | 0.40^***^ | — |  |  |  |  |  |  |  |  |  |  |  |  |  |
| **3. Centrality of work** | 0.40^***^ | 0.44^***^ | — |  |  |  |  |  |  |  |  |  |  |  |  |
| **4. Wasted time** | 0.34^***^ | 0.43^***^ | 0.41^***^ | — |  |  |  |  |  |  |  |  |  |  |  |
| **5. Anti-leisure** | 0.16^**^ | 0.24^***^ | 0.34^***^ | 0.15^**^ | — |  |  |  |  |  |  |  |  |  |  |
| **6. Delay of gratification** | 0.22^***^ | 0.40^***^ | 0.25^***^ | 0.31^***^ | 0.08 | — |  |  |  |  |  |  |  |  |  |
| **7.Self-reliance** | 0.10 | 0.11^*^ | 0.27^***^ | 0.25^***^ | -0.09 | -0.06 | — |  |  |  |  |  |  |  |  |
| **8. Morality/Ethics** | 0.15^**^ | 0.02 | 0.28^***^ | 0.19^***^ | -0.04 | 0.07 | 0.45^***^ | — |  |  |  |  |  |  |  |
| **9. MWEP** | 0.44^***^ | 0.68^***^ | 0.75^***^ | 0.68^***^ | 0.43^***^ | 0.52^***^ | 0.47^***^ | 0.47^***^ | — |  |  |  |  |  |  |
| **10. Amotivation** | 0.05 | 0.06 | -0.06 | -0.02 | -0.01 | -0.05 | -0.03 | -0.16^**^ | -0.06 | — |  |  |  |  |  |
| **11. External regulation** | 0.11^*^ | 0.19^***^ | -0.01 | 0.16^**^ | -0.04 | 0.08 | 0.17^***^ | 0.04 | 0.15^**^ | -0.10 | — |  |  |  |  |
| **12. Introjection** | 0.30^***^ | 0.36^***^ | 0.43^***^ | 0.25^***^ | 0.20^***^ | 0.22^***^ | 0.12^*^ | 0.16^**^ | 0.44^***^ | 0.03 | 0.21^***^ | — |  |  |  |
| **13. Identification** | 0.20^***^ | 0.28^***^ | 0.37^***^ | 0.24^***^ | 0.12^*^ | 0.14^**^ | 0.14^**^ | 0.14^**^ | 0.36^***^ | 0.04 | 0.25^***^ | 0.67^***^ | — |  |  |
| **14. Integration** | 0.20^***^ | 0.31^***^ | 0.48^***^ | 0.17^***^ | 0.23^***^ | 0.16^***^ | 0.04 | 0.15^**^ | 0.39^***^ | 0.04 | 0.04 | 0.65^***^ | 0.76^***^ | — |  |
| **15. Intrinsic motivation** | 0.23^***^ | 0.34^***^ | 0.50^***^ | 0.23^***^ | 0.23^***^ | 0.24^***^ | 0.09 | 0.25^***^ | 0.47^***^ | -0.11^*^ | 0.09 | 0.70^***^ | 0.61^***^ | 0.70^***^ | — |
| **16. Work self-determination index** | 0.13^**^ | 0.20^***^ | 0.48^***^ | 0.15^**^ | 0.22^***^ | 0.18^***^ | 0.02 | 0.24^***^ | 0.37^***^ | -0.40^***^ | -0.23^***^ | 0.51^***^ | 0.60^***^ | 0.77^***^ | 0.82^***^ |

MWEP – Multidimensional work ethic profile,

****p* < .001; ***p* < .01; **p* < .05.
