# Supplementary material for: Relationships between work ethic and motivation to work from the point of view of the self-determination theory
Source: PLoS One. 2021 Jul 1;16(7):e0253145. doi: 10.1371/journal.pone.0253145 (PMC8248600; doi:10.1371/journal.pone.0253145)
Supplement: S3 Table — Results of CCA for two canonical functions: canonical correlations, loadings, shared variance and redundancy analysis of independent and dependent canonical variates. ***p < .001. (DOCX) [file pone.0253145.s003.docx]

**Table 3. Work ethic and motivation. Results of CCA for two canonical functions: canonical correlations, loadings, shared variance and redundancy analysis of independent and dependent canonical variates**

| **Independent variables** | **Canonical loadings of independent variates** | |
| --- | --- | --- |
|  | U1 | U2 |
| **1. Work as moral obligation** | 0.412 | 0.482 |
| **2. Hard work** | 0.585 | 0.619 |
| **3. Centrality of work** | 0.920 | 0.028 |
| **4. Wasted time** | 0.362 | 0.531 |
| **5. Anti-leisure** | 0.448 | -0.102 |
| **6. Delay of gratification** | 0.405 | 0.239 |
| **7.Self-reliance** | 0.098 | 0.532 |
| **8. Morality/Ethics** | 0.395 | 0.005 |
| **Shared variance** | 0.253 | 0.156 |
| **Redundancy index** | 0.087 | 0.021 |
|  |  |  |
| **Canonical correlations** | 0.585^***^ | 0.362^***^ |
| **Overall redundancy (dependent set)** | 0.182 | |
| **Dependent variables** | **Canonical loadings of dependent variates** | |
|  | V1 | V2 |
| **9. Amotivation** | -0.136 | 0.287 |
| **10. External regulation** | 0.000 | 0.807 |
| **11. Introjection** | 0.782 | 0.451 |
| **12. Identification** | 0.620 | 0.382 |
| **13. Integration** | 0.858 | -0.003 |
| **14. Intrinsic motivation** | 0.931 | 0.122 |
| **Shared variance** | 0.436 | 0.183 |
| **Redundancy index** | 0.149 | 0.024 |

^***^*p* < .001
